# The Allergen Immunotherapy Adverse Events Registry: Setup & methodology of a European Academy of Allergy and Clinical Immunology taskforce project

**DOI:** 10.1002/clt2.12266

**Published:** 2023-06-08

**Authors:** Julijana Asllani, Dimitrios Mitsias, George Konstantinou, Alfred Priftanji, Mehmet Hoxha, Gerta Sinani, George Christoff, Dimitrov Zlatko, Michael Makris, Xenofon Aggelidis, Asja Stipic, Sanja Popovic‐Grle, Diana Deleanu, Vesna Tomic‐Spiric, Aleksandra Plavsic, Dilsad Mungan, Mitja Kosnik, Todor A. Popov, Nikolaos G. Papadopoulos, Moises Calderon, ADER study group, Gerta Sinani, Eris Mesonjesi, Esmeralda Shehu, Etleva Qirko, Anila Nano, Valbona Martini, Enkelejda Gjata, Aferdita Sinani, Jana Sinani, Sybi Musollari, Alketa Bakiri, Diana Qama, Erjola Piluri, Fatmira Xhixha, Gilda Xhoxhi, Mirela Hitaj, Mehmet Hoxha, Alfred Priftanji, Dimitrov Zlatko, George Christoff, Silviya Novakova, Katya Noleva, Plamen Yakovliev, Ted Popov, Mirjana Turkalj, Damir Erceg, Asja Stipic, Mira Pevec, Branko Pevec, Sanja Popovic‐Grle, Nikos Papadopoulos, Michael Makris, George Konstantinou, Dimitrios Mitsias, Xenofon Aggelidis, Christina Ciobanum, Ioana Agache, Florin Dan Popescu, Irina Bukur, Ierima Augustin, Dianna Deleanu, Adriana Muntean, Vesna Tomic‐Spiric, Rajica Stosovic, Mirjana Bogic, Dragana Tadic, Aleksandra Plavsic, Mitja Kosnik, Zeynep Mısırlıgil, Dilsad Mungan, Moises A. Z. Calderon

**Affiliations:** ^1^ University of Medicine Tirana Tirana Albania; ^2^ Allergy and Asthma Medical Clinic Tirana Albania; ^3^ Allergy Department 2nd Pediatric Clinic University of Athens Athens Greece; ^4^ Department of Allergy and Clinical Immunology 424 General Military Training Hospital Thessaloniki Greece; ^5^ University Hospital Center “Mother Teresa” Tirana Albania; ^6^ Internal Medicine Department Elbasan Regional Hospital Elbasan Albania; ^7^ Medical University‐Sofia Faculty of Public Health Sofia Bulgaria; ^8^ Acibadem City Clinic Tokuda Hospital Sofia Bulgaria; ^9^ Immunotherapy Outpatient Clinic Allergy Unit 2nd Department Dermatology and Venereology National and Kapodistrian University of Athens University General Hospital ‘Attikon’ Athens Greece; ^10^ Special Hospital for Pulmonary Diseases Zagreb Croatia; ^11^ Clinical Department for Lung Diseases Jordanovec University Hospital Center Zagreb Croatia; ^12^ University Medicine and Pharmacy Iuliu Hatieganu, IRGH, Allergy Cluj‐Napoca Romania; ^13^ Expertise Centre for Immunology & WAO Centre of Excellence Cluj‐Napoca Romania; ^14^ Clinic of Allergology and Immunology University Clinical Center of Serbia Belgrade Serbia; ^15^ Faculty of Medicine University of Belgrade Belgrade Serbia; ^16^ Department of Allergy and Immunology Ankara University School of Medicine Ankara Turkey; ^17^ University Clinic of Respiratory and Allergic Diseases Golnik Slovenia; ^18^ University Hospital “Sv Ivan Rilski” Sofia Bulgaria; ^19^ Lydia Becker Institute of Immunology and Inflammation University of Manchester Manchester UK; ^20^ Section of Allergy and Clinical Immunology Imperial College London‐NHLI London UK

To the Editor,

Over the last decades, systematic reviews and meta analyses of multiple double‐blind randomized placebo‐controlled trials have demonstrated the efficacy and safety of both subcutaneous (SCIT) and sublingual (SLIT) allergen immunotherapy (AIT) for respiratory and insect venom allergy.^1^ However, there is a wide range of AIT preparations used around the globe, and AEs are not always uniformly recorded, limiting the capability to draw safe conclusions or compare reports. Though various clinical development programs^2^ have generated substantial knowledge on different AIT preparations, they have been conducted in pre‐defined clinical conditions, not reflecting real‐life and therefore the results may not have generalized applicability.^3^ Very few pragmatic trials and observational studies have evaluated AIT safety in real‐life. Indicatively, Calderon et al.^4^ reported a total of 109 systemic reactions (SR) due to AIT recorded in 4316 patients in a prospective, longitudinal, “real‐life” web‐based survey. Another prospective study^5^ on SCIT safety in 581 patients reported immediate SR in 2.2%, delayed (7.4%), immediate local reactions (LR) (54.6%), while delayed in 56.1%.

Recently, the Respiratory Effectiveness Group highlighted the role of real‐world evidence (RWE) in (i) filling knowledge gaps, (ii) extending the implementation of findings from Randomized controlled trials in heterogeneous populations or healthcare systems, and (iii) providing evidence to generate clinical practice guidelines.^3^ Prospective, systematic RWE from registries are considered to provide a broadly applicable source of knowledge compared with retrospective approaches.^6^ However, there is still a lack of such registries in the allergy field.^7^ In this context, a new hierarchy of AIT RWE that ranks pragmatic trials and registry data at the highest level of evidence was proposed in a recent European Academy of Allergy and Clinical Immunology (EAACI) position paper.^6^

Considering these factors, a Task Force (TF) was created under the academic support of EAACI to develop an AIT Adverse Events Registry (ADER).

## 1 METHODOLOGY

Adverse Events Registry is a prospective, longitudinal, observational, multicenter, web‐based registry of “real‐life” AIT clinical practice across multiple countries. It has included centres from eight South‐Eastern European countries (Albania, Bulgaria, Croatia, Greece, Romania, Serbia, Slovenia and Turkey) and was academically supported by EAACI through a TF (AIT Adverse Events Registry Task Force). Our framework was based on the previous experience of the European Survey on Adverse Systemic Reactions in Allergen Immunotherapy.^8^ The TF was chaired by Nikolaos Papadopoulos and led by a Steering Committee (Nikolaos Papadopoulos, Moises Calderon, Ted Popov). The registry team, coordinated by Dimitrios Mitsias, included one to two National Coordinators per country, responsible for following local ethics requirements and recruiting physicians. No fees were provided for participant inclusion. The main objectives of the registry are (I) to assess systemic and local AEs occurring during regular clinical practice in real‐life settings, (II) to collect data and evaluate patient characteristics and AIT practice among countries (expected to differ substantially), (III) to identify independent factors associated with AE, and (IV) to use ADER as a model for AIT pharmacovigilance that could be subsequently expanded to other European countries. Based on the EASSI survey e–questionnaires,^8^ ADER developed three similar questionnaires to collect data (see Supplement).

## 2 RESULTS

Eligible subjects were adults and children attending regular consultations in real‐life clinical settings, presenting symptoms due to IgE mediated respiratory allergies documented by at least one positive skin prick test and/or specific IgE test to a panel of inhalant allergens. Those with verified Hymenoptera Venom Allergy and Venom Immunotherapy (VIT) courses to honey bees, common wasp and/or polistes wasp are also included. All patients are recruited in a prospective manner, on the same day they receive their first dose of a new AIT treatment, either SCIT or SLIT or VIT, and followed up until the end of the course. Systemic and local AEs are registered prospectively. A total of 2772 patients undergoing 3209 immunotherapy courses were retrieved up to May 2017 (Supplementary Table 1). Overall, 1019 systemic and local AEs were recorded in 330 patients. Additional information on the registry is described in the online supplement.

## 3 DISCUSSION

Registries are considered a powerful tool that can provide systematic collection of large amounts of data for a long period of treatment. Adverse Events Registry is the first AIT Registry for AE piloted in different countries in Europe that uses the harmonized MedDRA terminology^9^ (Table 1). These real‐life data will generate useful information that complement data from RCTs for decision‐making issues for doctors, patients, healthcare providers and policymakers. We aim to expand this Registry to more countries in Europe and possibly worldwide. The questionnaires gather data covering different safety aspects and other characteristics of AIT practice in a pragmatic, low‐effort manner. The inclusion of eight different countries leads to substantial heterogeneity (different populations, clinical settings, healthcare systems, AIT products and protocols), providing invaluable information on AIT safety. The registry is designed to collect all possible AEs during the course of a specific AIT in a real‐life setting. We are aware that this approach has a few shortcomings, especially in the case of SLIT (which is self‐delivered) or LR. However, the study attributes more weight to SR in AIT.

**TABLE 1:** Selected medical terms from the MedDRA dictionary used in this survey for recording systemic adverse reactions.

Systemic reactions
Abdominal pain
Angioedema (deeper swelling of skin/mucosa; single or multiple sites. Could not be well circumscribed & not itchy)
Asthma
Blood pressure decreased (suspicion of hypotension, but blood pressure not measured)
Bronchospasm
Chest discomfort
Chest tightness
Conjunctivitis allergic (eye swelling, pruritus, hyperaemia)
Cough
Diarrhoea
Dysphagia (swallowing difficulty/disorder)
Dysphonia (voice alteration)
Dyspnoea
Dizziness
Erythema (not at injection/application site but localized abnormal redness of the skin without any raised lesions)
Fatigue
Flushing (generalised flushing)
Generalised erythema
Headache
Hypotension (blood pressure measured systolic <90mmHg or >30% below baseline value)
Laryngeal oedema (objective glottic or vocal cord oedema)
Loss of consciousness
Nausea
Pruritus generalized
Rhinitis allergic (rhinorrhoea, sneezing, nasal congestion/itching)
Sensation of foreign body
Syncope (vasovagal, fainting)
Tachycardia (significant increase of the cardiac rhythm)
Urticaria (generalized)
Vomiting
Wheezing
Local reactions
Subcutaneous and Venom Immunotherapy
Local twinkling, itching, redness of the skin
Large local reaction of the skin
Sublingual Immunotherapy
Twinkling, itching, redness in oral cavity
Oedema in oral cavity

In conclusion, we consider ADER as a first effort to create a multinational network of centres practicing AIT and exchanging best practice parameters. We believe this is a step‐up in the progress of evidence needed to further support the optimal delivery of AIT. Such complex and factual monitoring may provide robust evidence of safety as well as help us to determine risk factors for AIT related AE.

## AUTHOR CONTRIBUTIONS

**Julijana Asllani**: data curation (equal); formal analysis (equal); writing original draft (lead). **Dimitrios Mitsias**: project administration (lead), methodology (equal), data curation (equal), and formal analysis (equal). **George Konstantinou**: data curation (equal); formal analysis (equal). **Todor A. Popov**: conceptualization (equal). **Nikolaos G. Papadopoulos**: conceptualization (equal); methodology (equal), funding acquisition (lead). **Moises Calderon**: conceptualization (equal); methodology (equal). **All authors**: Investigation (equal); writing‐review and editing (equal).

## CONFLICT OF INTEREST STATEMENT

Julijana Asllani, Alfred Priftanji, Mehmet Hoxha, Gerta Sinani, George Christoff, Dimitrov Zlatko, Michael Makris, Xenofon Aggelidis, Asja Stipic, Diana Deleanu, Vesna Tomic‐Spiric, Aleksandra Plavsic, Dilsad Mungan, Mitja Kosnik have no conflicts of interest to declare. Nikolaos G. Papadopoulos has received grants from Capricare, Nestle, Numil, Vianex and/or fees for consultancy from Abbott, Abbvie, Astra Zeneca, GSK, HAL, Medscape, Menarini/Faes Farma, Mylan, Novartis, Nutricia, OM Pharma, Regeneron/Sanofi in the last 3 years. Dimitrios Mitsias is or recently was a speaker for AstraZeneca, Gerolymatos International, Novartis, Sanofi, in the last 3 years. George Konstantinou is or recently was a speaker and/or advisor for and/or has received research funding from AstraZeneca, Chiesi, GSK, Menarini, Novartis, Pfizer, Sanofi, and Vianex in the last 3 years. All other authors do not declare any conflicts of interest.

## FUNDING INFORMATION

European Academy of Allergy and Clinical Immunology; Stallergenes

## Supporting information

Supplementary Material
