# Supplementary material for: The Allergen Immunotherapy Adverse Events Registry: Setup & methodology of a European Academy of Allergy and Clinical Immunology taskforce project
Source: Clin Transl Allergy. 2023 Jun 8;13(6):e12266. doi: 10.1002/clt2.12266 (PMC10249671; doi:10.1002/clt2.12266)
Supplement: Supplementary file 1 — Supplementary Material [file CLT2-13-e12266-s001.docx]

## Online Supplement

## Methodology additional information on the registry design and questionnaires.

Electronic Data Capture

The Research Electronic Data Capture (REDCap) online instrument was used to ensure safe uploading of the data. This is a browser-based, meta-data-driven electronic data capture (EDC) software solution and workflow methodology designed for rapid development and deployment of electronic data capture tools to support clinical and translational research databases [11]. All data uploaded were protected by an enhanced security system (Secure Sockets Layer, SSL), a protocol for encrypting information over the Internet.

Blinding and Data protection

To ensure anonymity, each practicing doctor was given an individual identification code to upload data. Only the National and General Coordinator have access to these codes in case any clarification regarding patient data was needed. Each NC has access only to the data from his respective country while full dataset access is allowed only to the General Coordinator and the researchers responsible for the collection and analysis of data. Also each patient is given a code that links him to the data by the participating doctor and he is the only one to have access to patients’ personal data.

Ethics

ADER complies with national and European ethical and regulatory requirements, including data protection. Each NC was responsible for obtaining study approval from their corresponding national or local independent Ethics Committee.

Questionnaires Design

Based on the EAASI survey e-questionnaires [8], the ADER’s working team created three similar questionnaires: i) Doctor’s Questionnaire (DQ), ii) Patient’s Questionnaire (PQ) and iii) Reaction’s Questionnaire (RQ). Most of the questions are designed to be answered in a compulsory manner. A beta-test system was performed before launching the Registry. The modified MedDRA classification used in the EASSI survey was selected to describe all adverse reactions [9]. Data on local reactions were also recorded. Therefore, short questions related to SCIT or SLIT local reactions were added.

i**) Doctor’s Questionnaire (DQ) (Additional file 1).**

The DQ is completed by the participating doctor and includes information about the doctor prescribing AIT, such as country of residence, medical specialty, clinical experience (in years), setting of practice (public or private), percentage of new patients prescribed to receive AIT in the previous year and approximate percentage of SCIT or SLIT treatments prescribed.

**ii) Patient’s Questionnaire (PQ) (Additional file 2).**

This questionnaire should be completed once per each new AIT course prescribed and initiated. It includes questions regarding patient’s profile: demographic data, past medical history, allergy history, allergic profile (skin prick tests, sIgE, and clinical significance), possible previous AIT courses [allergen(s), route and tolerance]. The main corpus of the PQ, will record data regarding the current AIT course: route (SCIT or SLIT), relevant allergen(s), type of allergen delivered (natural extract or allergoid for SCIT, drops or tablet for SLIT), type of adjuvant (e.g. aluminum, phosphate, tyrosine or none for SCIT), brand and vaccine name, initial phase schedule (i.e. conventional, cluster, rush or ultra-rush), months/year of AIT, possible premedication and personnel that delivers AIT. In case a patient undergoes two (or more) AIT courses, the PQ are filled for each course.

**iii) Adverse Reaction’s Questionnaire (RQ) (Additional file 3).**

This questionnaire was designed to record any adverse reaction, systemic or local, occurring in a patient receiving AIT, either SCIT or SLIT. The treatment phase when the reaction occurred (up-dosing or maintenance) and clinical symptoms of the AE (according to the MedDRA classification in case of systemic reaction) [9] are collected. Also, medication used to treat the AE, final outcome of the AE and possible treatment modification. Severity was estimated in a dual manner: I) according to Muller [10], and II) a 3-grade classification system: mild (symptoms that don't interfere with daily activities), moderate (strong symptoms that interfere regularly in daily activities) or severe (unacceptable symptoms that interfere considerably in daily activities) [8]. Seriousness was judged by the physician as low or high and co-factors influencing the AE were tracked. The RQ can be uploaded multiple times in case a patient undergoes an AE more than once. When an AIT course ends the physician fills an “End study form” with the date and the course is considered finished.

Harmonized MedDRA terminology

As a team decision, we agreed that all symptoms described as “systemic adverse reactions” will be reported using the harmonized, clinically validated, international medical MedDRA terminology [9]. The shortened list of terms used in the EASSI project was selected in our case [8] (Table 1). The use of MedDRA terminology is supported by different international agencies, including the European Medicines Agency (EMA), because it classifies adverse events information associated with the use of biopharmaceuticals and other medical products, allowing health authorities and industry to exchange and analyze data related to the safe use of medical products.

*Results*

Out of 2,813 treatments that were prescribed for respiratory allergies 1,245 were SCIT (44.3%) and 1,568 SLIT (55.7%). In 373 (13.4%) patients venom immunotherapy (n=396 courses) was prescribed (Supplementary Table 1). AE were recorded in 11.9% of the cases. Most adverse reactions (46.4%) occurred during VIT and SCIT for respiratory allergies (45%) compared to SLIT (8.6%). Most patients (70.2%) experienced mild reactions. One AIT treatment was prescribed in most patients (85.9%). Prescription rate of SCIT vs. SLIT, allergens, extracts and protocols differed among countries.

# References

8. Calderon MA, Rodríguez Del Río P, Vidal C, Just J, Pfaar O, Linneberg A, et al. (2014). "An EAACI "European Survey on Adverse Systemic Reactions in Allergen Immunotherapy (EASSI)": the methodology." Clin Transl Allergy 4: 22.

9. Medical Dictionary for Regulatory Activities Website. <http://www.meddra.org>

10. Mueller UR. Clinical presentation and pathogenesis. In: Mueller UR, editor. Insect sting allergy. Stuggart: Gustav Fischer; 1990. p. 33-65.

11. Harris PA, Taylor R, Thielke R, Payne J, Gonzalez N, Conde JG. Research electronic data capture (REDCap)--a metadata-driven methodology and workflow process for providing translational research. *J Biomed Inf*. 2009;42(2):377‐381. https://doi.org/10.1016/j.jbi.2008.08.010. https://www.project-redcap.org/

# Supplementary Table 1. Distribution of patients and AIT treatments by type and country.

|  | **Total** | **SCIT** | | **SLIT** | | **VIT** | |
| --- | --- | --- | --- | --- | --- | --- | --- |
|  | *Patients*  *n* | *Patients*  *n (%)* | *Treatments*  *n* | *Patients*  *n (%)* | *Treatments*  *n* | *Patients*  *n (%)* | *Treatments*  *n* |
| Albania | 601 | 429 (71.4) | 434 | 148 (24.6) | 148 | 24 (4) | 24 |
| Bulgaria | 743 | 122 (16.4) | 133 | 617 (83.1) | 625 | 4 (0.5) | 4 |
| Croatia | 164 | 35 (21.3) | 35 | 35 (21.3) | 35 | 94 (57.4) | 94 |
| Greece | 633 | 220 (35.7) | 345 | 323 (51) | 470 | 90 (14.2) | 109 |
| Romania | 78 | 44 (56.4) | 44 | 13 (16.6) | 13 | 21 (27) | 21 |
| Serbia | 319 | 57 (18) | 116 | 203 (63.6) | 249 | 59 (18.5) | 63 |
| Slovenia | 133 | 49 (36.8) | 60 | 26 (19.5) | 28 | 58 (43.6) | 58 |
| Turkey | 101 | 78 (77.2) | 78 | 0 | 0 | 23 (22.7) | 23 |
| Total | 2772 | 1034 | 1245 | 1365 | 1568 | 373 | 396 |

Questionnaires

# Doctor Questionnaire

Doctor Code ________________________________

1. Which is your country of residence? ☐Albania

☐Croatia

☐Hungary

☐ Romania

☐ Serbia

☐Fyrom

☐ Greece

☐ Turkey

☐ Bulgaria

☐ Slovenia

2. Which is /are your speciality/ties?

☐Allergy ☐Pulmonology ☐Dermatology ☐ENT

☐Immunology ☐ Pediatrics ☐GP ☐Other

Please specify ________________________________

**3. Could you please specify your experience in.....?**

Allergy (in years) ________________________________

Allergen Specific Immunotherapy (in years) ________________________________

4. What is the affiliation of the patients that you will include in this survey?

☐Public ☐Private

5. What is the approximate number of NEW patients ________________________________

with respiratory allergy that you see per year?

6. Approximately what percentage (%) of these NEW patients go into Allergen Immunotherapy?


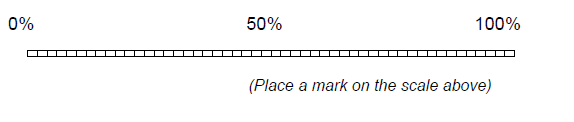


**7. How are your prescriptions, expressed in percentage (%), divided between both routes?**

Subcutaneous Immunotherapy ________________________________

Sublingual Immunotherapy ________________________________

Warning! The values you entered do not add to 100%!

# Patient Questionnaire

Study ID ________________________________

Select your Personal Doctor Code (email) ________________________________

________________________________

________________________________

Patient's date of birth ________________________________

Patient's residence country ☐Albania

☐Croatia

☐Hungary

☐Romania

☐Serbia

☐Fyrom

☐Greece

☐Turkey

☐Bulgaria

☐Slovenia

Patient's gender

☐Female ☐ Male

Patient's medical history(apart from allergic diseases)

☐Cardiovascular disease

☐Previous episode of anaphylaxis

☐Mastocytosis

☐No relevant medical history

☐Drug intake

☐Gastritis/ulcer

☐Neuro/Psychiatric disorders

☐Skin disorders (not allergic)

☐GI tract disorders

☐Rheumatic disorders

☐Respiratory disorders (not allergic)

☐Other

Please specify drug intake ☐β-blockers ☐ACE inhibitors

☐other

Please specify other drug intake ________________________________

Please specify other medical history ________________________________

Patient's allergy history

☐ Atopic Dermatitis

☐ Asthma

☐Conjuctivitis

☐ Rhinitis

☐ Chronic Urticaria

☐ Drug allergy

☐ Food allergy

☐ Hymenoptera allergy

☐ Other

Please specify ________________________________

Is the patient having any active treatment for allergy ?

☐Yes ☐No

Patient's use of medication for allergy

☐ Inhaled beta-agonist

☐ Inhaled corticosteroids

☐ Nasal corticosteroids

☐ Nasal antihistamines

☐ Long Acting Beta Agonists (LABA)

☐Antileukotrienes

☐ Anticholinergics

☐ Antihistamines

☐ Sodium cromoglycate

☐ Ocular preparations

☐ None

☐ Other

Please specify other medication used ________________________________

**Patient's allergy profile: Tick where suitable for sensitisation diagnosis (SPT and/or specificIgE) and if clinically significant**

**SPT and/or ID for hymenoptera**

|  | SPT > 3mm | sIgE> 0.7 kU/L | Clinicallysignificant | Notperformed |
| --- | --- | --- | --- | --- |
| Pollen | ☐ | ☐ | ☐ | ☐ |
| Mites | ☐ | ☐ | ☐ | ☐ |
| Food | ☐ | ☐ | ☐ | ☐ |
| Epithelia | ☐ | ☐ | ☐ | ☐ |
| Molds | ☐ | ☐ | ☐ | ☐ |
| Hymenoptera | ☐ | ☐ | ☐ | ☐ |
| Latex | ☐ | ☐ | ☐ | ☐ |

Is the patient sensitised to pollen?

☐Yes ☐No

Indicate which pollens the patient is sensitised to:

☐ Grass

☐ Birch

☐ Olive

☐ Ash

☐ Plane tree

☐ Cupressus

☐ Hazel

☐ Alder

☐Mugwort

☐ Ragweed

☐Parietaria

☐Plantago

☐ Chenopodium

☐ Saltwort

☐ Bermuda

☐ Other

Please specify ________________________________

Has the patient received any Allergen Specific Immunotherapy (AIT) before? Not applicable in case of two parallel AIT regimens.

☐Yes ☐No

Which route

☐ Sublingual ☐ Subcutaneous ☐ Both

Its composition

☐Grasses ☐Trees ☐Weeds ☐Dust Mites ☐Epithelia Molds ☐Hymenoptera☐Latex ☐ Food

Its tolerance

☐ Very Good (no local, no systemic reactions)

☐ Good (only local reactions)

☐ Moderate (local and systemic reactions or systemic reactions only)

☐Poor (one or more severe systemic reactions)

☐ Very Poor (any anaphylactic shock)

For the previous SLIT performed by the patient, indicate its composition:

☐Grasses ☐Trees ☐Weeds ☐Dust Mites ☐Epithelia Molds ☐Hymenoptera☐ Latex ☐ Food

For the previous SLIT performed by the patient, indicate its tolerance:

☐ Very Good (no local, no systemic reactions)

☐ Good (only local reactions)

☐ Moderate (local and systemic reactions or systemic reaction only)

☐ Poor (one or more severe systemic reactions)

☐ Very Poor (any anaphylactic shock)

For the previous SCIT performed by the patient, indicate its composition:

☐Grasses ☐Trees ☐Weeds ☐Dust Mites ☐Epithelia Molds ☐Hymenoptera☐ Latex ☐ Food

For the previous SCIT performed by the patient, indicate its tolerance:

☐ Very Good (no local, no systemic reactions)

☐ Good (only local reactions)

☐ Moderate (local and systemic reactions or systemic reaction only)

☐ Poor (one or more severe systemic reactions)

☐ Very Poor (any anaphylactic shock)

For the current AIT, indicate the date(only year) ________________________________of onset of the allergic disease/first reaction

Date when the first dose of the current AIT was ________________________________

administered

Composition of current AIT

☐ Grass

☐ Bermuda

☐ Olive

☐ Birch

☐Cuprasseceae

☐ Mites

☐ Alternaria (other molds are excluded )

☐Parietaria

☐ Cat epithelia

☐ Dog

☐ Alder

☐Mugwort

☐ Hazel

☐ Ragweed

☐Plantago

☐ Tree mix

☐ Tree other

☐ Weed mix,

☐ Horse

☐ Honey Bee venom

☐ Wasp venom

☐Polistes venom

☐ Other

Please specify ________________________________

Other percentage


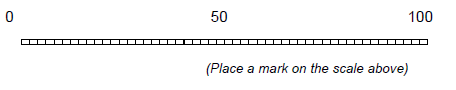


Olive percentage


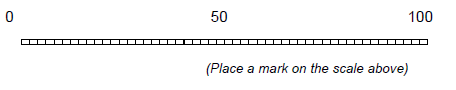


Polistes venom percentage


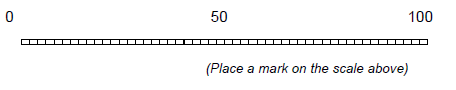


Wasp venom percentage


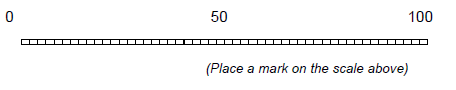


Honey Bee venom percentage


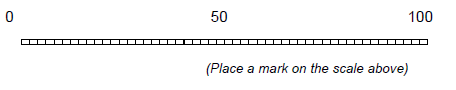


Dog percentage


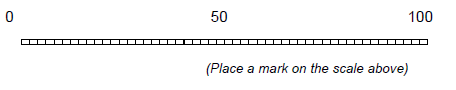


Cat - epithelia percentage


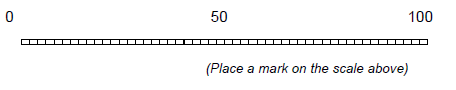


Parietaria percentage


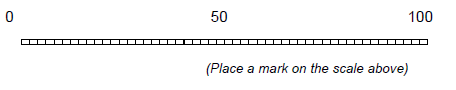


Alternaria percentage


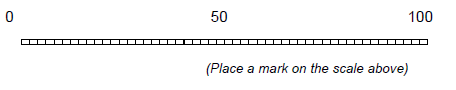


Mites percentage


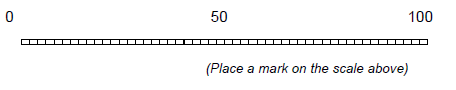


Cuprasseceae percentage


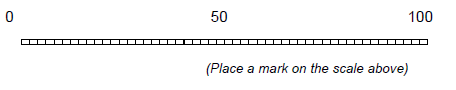


Birch percentage


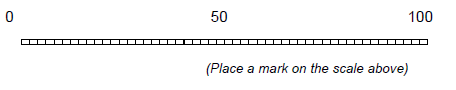


Bermuda percent


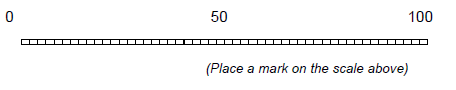


Grass percentage


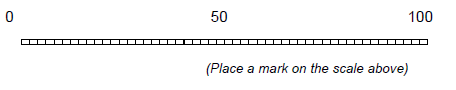


Alder percentage


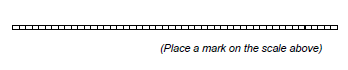


Mugwort percentage


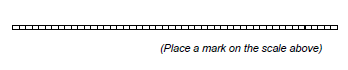


Hazel percentage


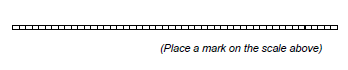


Ragweed percentage


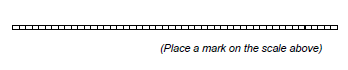


Plantago percentage


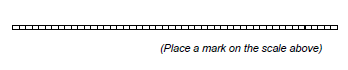


Tree mix percentage


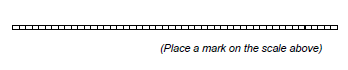


Tree other percentage.


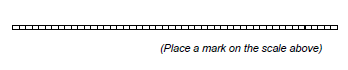


Weed mix percentage


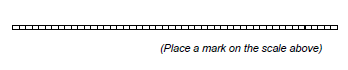


Horse percentage


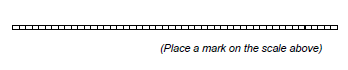


Please specify manufacturers ☐Allergopharma

☐ Allergy Therapeutics

☐ ALK- Abello

☐Anallergo

☐ BIAL- Aristegui

☐ HAL

☐Leti

☐Lofarma

☐Stallergenes

☐Torlak

☐Bul Bio

☐Inmunotec

☐ Other

Please specify other manufacturer ________________________________

Please specify Brand Name

☐Allergopharm/Novo-Helisen depot ☐Allergopharm SLIT/Allergodrop ☐Allergovit☐Acaroid ☐Allerslit forte

Please specify Brand Name

☐Αlutek ☐Alxoid ☐Clustek ☐Oraltek

Please specify Brand Name

☐Pollinex4 ☐Tyrosin TU ☐Oralvac ☐Venom ATL

Please specify Brand Name

☐ALK Specific ☐Alutard SQ ☐Avanz ☐SLITone ☐Grazax☐Pharmalgen

Please specify Brand Name

☐SLIT venom

☐SLIT 3-4-5

☐SLIT with bacterial adj.

☐SCIT venom aq.

☐SCIT venom Tyrosin

☐SCIT allergoid Tyrosin

☐SCIT Tyrosin

Please specify Brand Name

☐ERB ☐Alberi

Please specify Brand Name

☐Depothal ☐Purethal ☐Sublivac ☐Venomhal

Please specify Brand Name

☐Depigoid ☐TOLforte

Please specify Brand Name

☐Depot ☐Lais ☐Allerkin

Please specify Brand Name

☐Alustal ☐Phostal ☐Epi ☐Staloral ☐Staloral 300 ☐Oralair☐Alyostal

Who will administrate AIT in this patient?

☐Physician ☐ Nurse ☐Self administration ☐Other

Please specify ________________________________

What is the administration route in the current AIT?

☐Subcutaneous ☐Sublingual

Which type of extract?

☐Natural ☐Allergoid

Which type of adjuvant?

☐none ☐aluminium hydroxide ☐MPL ☐calcium phosphate ☐tyrosine ☐other

Please specify other adjuvant ________________________________

Which type of formulation?

☐Drops ☐Tablets

Type of up-dosing schedule (SCIT)

☐Conventional ☐Cluster ☐Rush ☐Ultrarush

Type of up-dosing schedule (SLIT)

☐Conventional (according to manufacturer) ☐Rush

State the frequency of doses given during maintenance:

☐Daily ☐ Each 48 hours ☐Weekly ☐Every two weeks ☐Monthly (4-6 week) ☐up to 6 weeks

Current AIT is followed to which schedule?

☐Perennial ☐ Co- seasonal ☐Pre- seasonal ☐Pre/Co - seasonal

How many months of AIT is planned to be given in a ________________________________

year?

Use of premedication for current AIT (initial phase)?

☐Antihistamines ☐Corticosteroids ☐None ☐Other

Please specify ________________________________

# Reactions Questionnaire

Study ID ________________________________

Select your Personal Doctor Code (email) ________________________________

________________________________

________________________________

Patient's date of birth ________________________________

Patient's gender

☐ Female ☐ Male

At which phase was the Adverse Reaction reported?

☐Up- dosing(increasing dose)☐Maintenance (4-6 weeks)☐maintenance>6 weeks

Indicate the date of the reported adverse reaction. ________________________________

Type of Adverse Reaction according to the MedDRA Classification for Systemic Reactions:

☐Abdominal pain

☐Angioedema (Deeper swelling of skin/mucosa; single or multiple sites.Could not be well circumscribed & not itchy)

☐Asthma

☐Blood pressure decreased (Suspicion of hypotension, but blood pressure not measured)

☐Bronchospasm

☐Chestdiscomfort

☐Chesttightness

☐Conjuctivitis allergic ( Eye swelling, pruritus, hyperaemia)

☐Cough

☐Diarrhoea

☐Dysphagia (Swallowing difficulty / disorder)

☐Dysphonia ( Voice alteration)

☐Dyspnoea

☐Dizziness

☐Erythema (Not at injection/application site but localised abnormal redness of the skin without any raised lesions)

☐Fatigue

☐Flushing (Generalised flushing)

☐Generalised erythema

☐Headanche

☐Hypotension (Blood pressure measured Systolic < 90mmHg or >30% below baseline value)

☐Laryngeal oedema (Objective glottic or vocal cord oedema)

☐Loss of consciousness

☐Pruritus generalized

☐Rhinitis allergic (Rhinorrhoea,Sheezing,nasal congestion/itching)

☐Sensation of foreign body

☐Syncope (Vasovagal, fainting)

☐Tachycardia (Significant increase of the cardiac rhythm)

☐Urticaria (local reaction)

☐Urticaria(generalized)

☐Vomiting

☐Wheezing

☐Local reaction

☐Other

Please specify other ________________________________

What is the administration route in the current AIT?

☐ Subcutaneous ☐ Sublingual

☐ No local reactions

Local reactions

☐ Local twinkling, itching, redness of the skin

☐ Large local reaction of the skin

Local reactions

☐ Twinkling, itching, redness in oral cavity

☐Oedema in oral cavity

Please record medication used to treat the reaction

☐ Adrenaline / epinephrine intramuscular

☐ Adrenaline / epinephrine intravenous

☐ Adrenaline / epinephrine subcutaneous

☐ Antihistamines intramuscular

☐ Antihistamines intravenous

☐ Antihistamines oral

☐ Corticosteroids intravenous

☐ Corticosteroids oral

☐ Beta 2 agonists

☐ Cardiopulmonary resuscitation

☐ Fluid (saline)

☐ Fluid (expanders)

☐ Glucagon

☐ Oxygen

☐ Transfer to intensive care unit treatment

☐Vasopressing

☐ None

☐ Other

Please specify other ________________________________

Severity of reaction

☐ Mild (Symptoms that don't interfere with daily activities)

☐ Moderate (Strong symptoms that interfere regularly in daily activities)

☐ Severe (Unacceptable symptoms that interfere considerably in daily activities)

Severity of reaction according to Muller classification

☐ΝΑ

☐ Grade I Generalized urticaria itching, malaise and anxiety

☐ Grade II Any of the above plus two or more of the following: angioedema, chest constriction, nausea, vomiting, diarrhea, abdominal pain, dizziness

☐Grade III Any of the above plus two or more of the following: dyspnea, wheezing, stridor,dysarthria,hoarseness,weakness,confusion, feeling of impending disaster

☐ Grade IV Any of the above plus two or more of the following: fall in blood pressure, collapse,loss ofconsciousness,incontinence,cyanosis

Seriousness of reaction

☐Low ☐High

Did you modify the schedule?

☐ Yes ☐ No

Which modification did you apply?

☐Lower dose

☐Change initial phase protocol

☐Change Company, regimen

☐Add omalizumab

☐discontinue IT

Causality of Adverse Reaction (relationship to administration of AIT):

☐Yes Possible (causal relationship to AIT is reasonable and cannot be dismissed)

☐No Unlikely (likely related to another etiology than AIT; i.e other drugs or underlying disease)

Have you identified any cofactor(s) influencing the Adverse Reaction?

☐Exercise

☐Anti-inflammatory drugs

☐Infection

☐Exposure to allergen(s)

☐Uncontrolled asthma

☐Oral mucosa problem

☐None

☐Other

Please specify other cofactor ________________________________

Elapsed time from last Allergen AIT administration to _____________________________

the Systemic Adverse Reaction

The systemic Adverse Reaction Lasted _____________________________

Outcome of Systemic Adverse Reaction ☐Resolution ☐Hospitalization

☐Death
